# Rapid conversion of porcine pluripotent stem cells into macrophages with chemically defined conditions

**DOI:** 10.1016/j.jbc.2023.105556

**Published:** 2023-12-12

**Authors:** Xiaolong Wu, Yu Ni, Wenhao Li, Bin Yang, Xinchun Yang, Zhenshuo Zhu, Juqing Zhang, Xiaojie Wu, Qiaoyan Shen, Zheng Liao, Liming Yuan, Yunlong Chen, Qian Du, Chengbao Wang, Pentao Liu, Yiliang Miao, Na Li, Shiqiang Zhang, Mingzhi Liao, Jinlian Hua

**Affiliations:** 1 College of Veterinary Medicine, Shaanxi Centre of Stem Cells Engineering and Technology, Northwest A&F University, Yangling, Shaanxi, China; 2 College of Life Sciences, Northwest A&F University, Yangling, Shaanxi, China; 3 School of Biomedical Sciences, Li Ka Shing Faculty of Medicine, Stem Cell and Regenerative Medicine Consortium, The University of Hong Kong, Pokfulam, Hong Kong, China; 4 Institute of Stem Cell and Regenerative Biology, College of Animal Science and Veterinary Medicine, Huazhong Agricultural University, Wuhan, China

**Keywords:** rapid conversion, macrophages, porcine pluripotent stem cells, hematopoiesis, TGF-β signaling pathway

## Abstract

A renewable source of porcine macrophages derived from pluripotent stem cells (PSCs) would be a valuable alternative to primary porcine alveolar macrophages (PAMs) in the research of host–pathogen interaction mechanisms. We developed an efficient and rapid protocol, within 11 days, to derive macrophages from porcine PSCs (pPSCs). The pPSC-derived macrophages (pPSCdMs) exhibited molecular and functional characteristics of primary macrophages. The pPSCdMs showed macrophage-specific surface protein expression and macrophage-specific transcription factors, similar to PAMs. The pPSCdMs also exhibited the functional characteristics of macrophages, such as endocytosis, phagocytosis, porcine respiratory and reproductive syndrome virus infection and the response to lipopolysaccharide stimulation. Furthermore, we performed transcriptome sequencing of the whole differentiation process to track the fate transitions of porcine PSCs involved in the signaling pathway. The activation of transforming growth factor beta signaling was required for the formation of mesoderm and the inhibition of the transforming growth factor beta signaling pathway at the hematopoietic endothelium stage could enhance the fate transformation of hematopoiesis. In summary, we developed an efficient and rapid protocol to generate pPSCdMs that showed aspects of functional maturity comparable with PAMs. pPSCdMs could provide a broad prospect for the platforms of host-pathogen interaction mechanisms.

Porcine alveolar macrophages (PAMs) are one of the important immune cells in organisms, serving as the first immune barrier for animal pathogens to infect the lungs; they also are the main host cells of African swine fever virus and porcine respiratory and reproductive syndrome virus (PRRSV) (1, 2, 3, 4). Therefore, PAMs are an ideal platform for studying host–pathogen interaction mechanisms. However, primary PAMs cannot be easily stably expanded *in vitro* because of the lack of development mechanism and biological characteristics of PAMs. The differences in genetic background and immunological status also result in serious batch-to-batch variation among isolated PAMs, and genetic modification is challenging for isolated PAMs, which seriously hampers the study of host–pathogen interaction mechanisms. The immortalized PAMs, such as WSL and 3D4-21, lose some of macrophage characteristics (5, 6, 7). Therefore, WSL and 3D4-21 are not ideal cell models for studying host–pathogen interaction mechanisms. Pluripotent stem cells (PSCs) can be steadily expanded, genetically modified, and differentiated into macrophages *in vitro* (8, 9, 10, 11, 12, 13, 14, 15, 16, 17). Therefore, macrophages derived from PSCs provide a broad prospect for the platforms of host–pathogen interaction mechanisms.

Mammalian hematopoiesis is a complex biological process involving three waves of temporally and spatially distinct hematopoiesis. The first wave occurs in the yolk sac and generates primitive macrophages, which migrate to the brain and form microglia (18, 19, 20, 21, 22). The second wave also occurs in the yolk sac and gives rise to multipotent progenitors, including erythromyeloid progenitor cells (EMPs); this process is independent of the *MYB* gene. EMPs migrate to the fetal liver and colonize various tissues (except the brain) to generate tissue-resident macrophages at developmental stages (19, 23, 24, 25). The third wave mainly occurs in the aorto-gonadal-membrane, where hematopoietic endothelium (HE) goes the endothelial-to-hematopoietic transition process to generate hematopoietic progenitor cells (HPCs); this process is *MYB*-dependent (26, 27, 28). HPCs are the main source of peripheral blood mononuclear-derived macrophages (PBMCs) and bone marrow-derived macrophages (BMDMs) (29, 30, 31, 32). According to previous reports, PSC-derived macrophages are similar to tissue-resident macrophages, as they undergo *MYB*-independent myeloid differentiation (13, 33, 34, 35). PSC-derived macrophages *in vitro* can mimic the features of tissue-resident macrophages *in vivo*.

The transforming growth factor beta (TGF-β) signaling pathway plays a vital role in hematopoiesis. However, its function remains unclear. During the formation of the human and porcine hematopoietic mesoderm (ME), PSCs can be differentiated into ME with the addition of basic morphogenetic protein 4 (BMP4), CHIR99021, and activin A (8, 9, 36, 37, 38). However, hematopoietic ME can also be differentiated from PSCs with the addition of BMP4 or CHIR99021 (10, 11, 39, 40, 41, 42). Whether the TGF-β signaling plays a vital role in ME formation remains unclear. During the formation of HE, whether the TGF-β signaling pathway is inhibited or not is also a critical issue. The inhibition of the TGF-β signaling pathway is required in the formation of HE differentiation (8, 9, 28, 38, 43). However, some experiments have shown that the activation of the TGF-β signaling pathway is required in the formation of HE differentiation (10, 40, 41), and the inhibition of the TGF-β signaling pathway can block hematopoiesis (36, 37). Therefore, the role of the TGF-β signaling pathway in porcine hematopoiesis needs to be further explored.

As previously reported, porcine PSCs were differentiated into macrophages by the suspension of embryoid bodies (EBs) (42). However, this protocol presented a long differentiation time of around 20 days and could not clearly define the various cell types, like HE and EMPs, during the differentiation process. Here, we described a protocol that allowed the production of EMP-like cells that could be further differentiated toward mature pPSC-derived macrophages (pPSCdMs) in only 11 days with chemically defined conditions. We comprehensively analyzed the global gene expression by total RNA-seq at different stages during pPSCdMs differentiation. We also found that TGF-β signaling pathway plays a dual role in differentiation. The formation of hematopoietic ME required the activation of the TGF-β signaling pathway, and the silence of the TGF-β signaling pathway at the HE stage would enhance the hematopoiesis. Finally, we compared pPSCdMs and PAMs *via* functional assays, including endocytosis, phagocytosis, PRRSV infection, and stimulation of bacterial lipopolysaccharide (LPS). In summary, the pPSCdMs express macrophage-surface markers and specific transcription factors. Moreover, the pPSCdMs have also the function of macrophages, similar to PAMs, and are ideal cell models for studying host-pathogen interaction mechanisms.

## Results

### Differentiation of macrophages from porcine EPSCs

**Figure 1:**
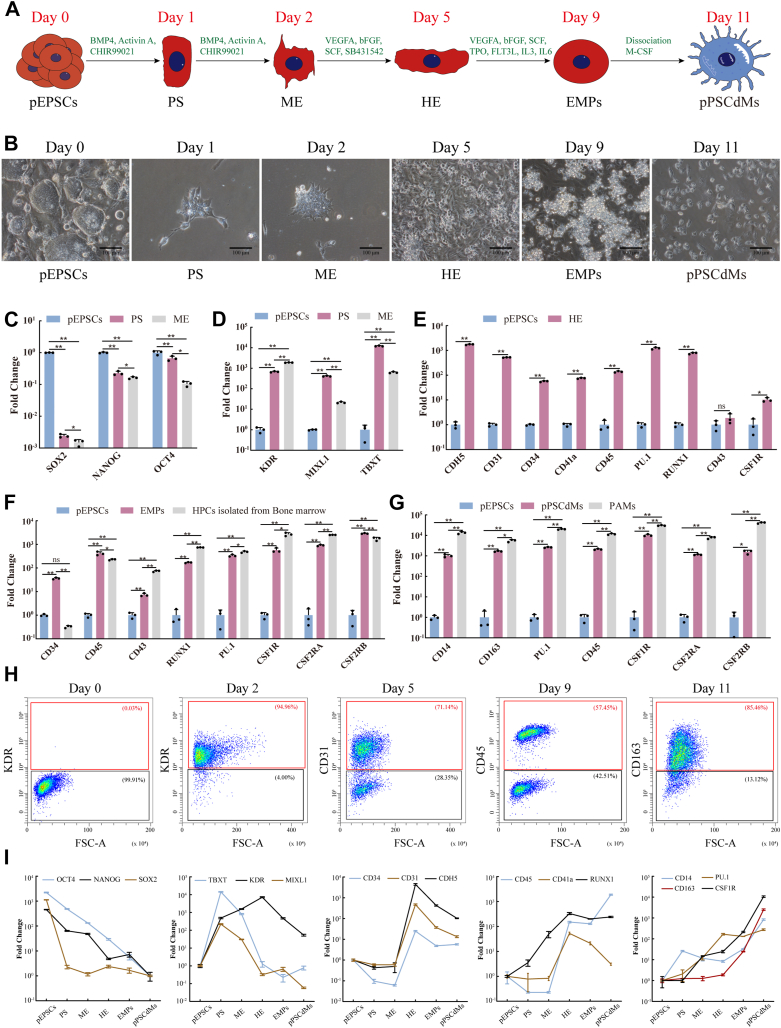
**Differentiation of macrophages from porcine EPSCs.***A*, schematic overview of macrophages differentiation protocols from pEPSCs. *B*, representative bright-field images were shown for the different morphologies generated on day 0 (undifferentiated pEPSCs), day 1 (PS), day 2 (ME), day 5 (HE), day 9 (EMPs), and day 11 (pPSCdMs). Scale bars represent 100 μm. *C*, the expression levels of pluripotent-related genes in pEPSCs, PS, and ME were detected by RT-qPCR. Data indicate mean ± s.d. The *p* values were calculated by using two-tailed *t*-tests. n = 3. *D*, the expression levels of PS-related and mesodermal genes in pEPSCs, PS, and ME were detected by RT-qPCR. Data indicate mean ± s.d. The *p* values were calculated by using two-tailed *t*-tests. n = 3. *E*, the expression levels of HE-related genes in pEPSCs and HE were detected by RT-qPCR. Data indicate mean ± s.d. The *p* values were calculated by using two-tailed *t*-tests. n = 3. *F*, the expression levels of EMP-related genes in pEPSCs, EMPs, and HPCs isolated from porcine bone marrow were detected by RT-qPCR. Data indicate mean ± s.d. The *p* values were calculated by using two-tailed *t*-tests. n = 3. *G*, the expression levels of macrophage-related genes in pEPSCs, pPSCdMs, and PAMs were detected by RT-qPCR. Data indicate mean ± s.d. The *p* values were calculated by using two-tailed *t*-tests. n = 3. *H*, FACS analysis of stage-specific markers on day 0, day 2, day 5, day 9, and day 11 during differentiation from pEPSCs. The percentages of positive populations were shown in *red*. *I*, RT-qPCR expression profile analysis of every differentiation stage for pluripotency markers (*POU5F1*, *SOX2*, and *NANOG*), PS markers (*TBXT* and *MIXL1*), ME marker (*KDR*), HE markers (*CD31*, *CD34*, *RUNX1* and *CDH5*), EMPs markers (*RUNX1*, *CD45*, and *ITGA2B*), and pPSCdMs markers (*CSF1R*, *CD14*, *CD163*, and *PU.1*). Data indicate mean ± s.d. n = 3. EMPs, erythromyeloid progenitor cells; EPSCs, expanded potential stem cells; FACS, fluorescence-activated cell sorting; HE, hematopoietic endothelium; HPCs, hematopoietic progenitor cells; ME, mesoderm; PAMs, porcine alveolar macrophages; pEPSCs, porcine expanded potential stem cells; pPSCdMs, pPSC-derived macrophages; PS, primitive streak; RT-qPCR, quantitative reverse transcription PCR.

**Supporting Figure S1:**
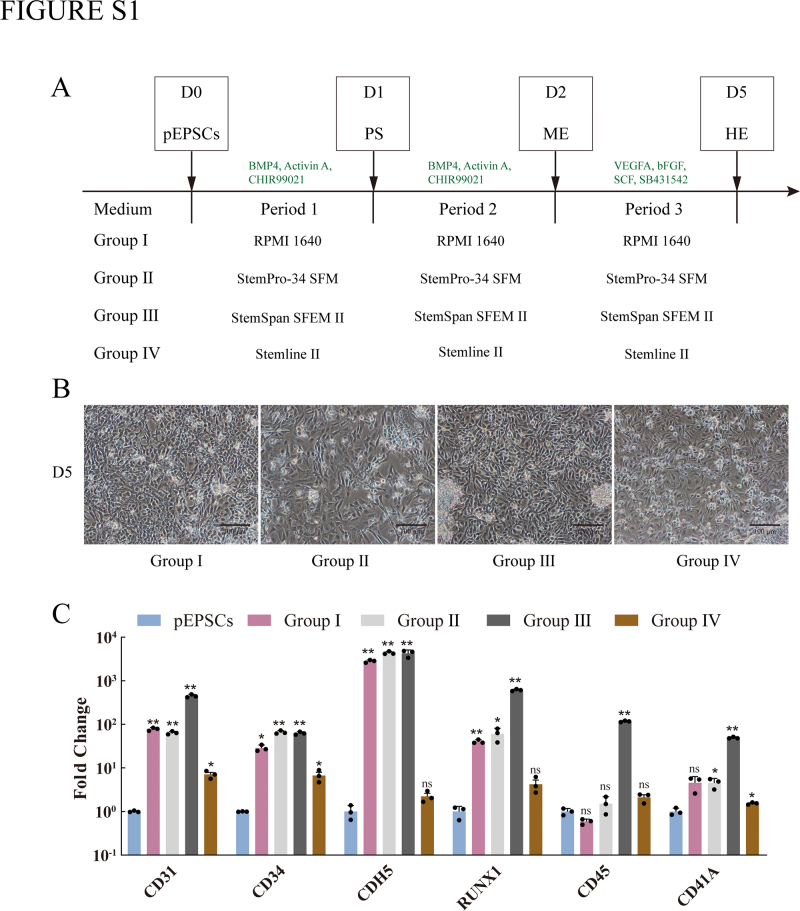
*A*, schematic overview of the different basal media were used for hematopoietic differentiation. *B*, representative bright-field images were shown for HE derived from different basal media. Scale bars represent 100 μm. *C*, the expression levels of hematopoietic genes in different groups were detected by RT-qPCR. Data indicate mean ± s.d. The *p* values were calculated by using two-tailed *t*-tests. n = 3.

Porcine expanded potential stem cells (pEPSCs) were cultured on mitotically inactivated sandos inbred mice 6-thioguanine-resistant, ouabain-resistant cells (STO) feeder layers in pEPSCs medium (44) as reported previously and differentiated into macrophages by using protocols adapted from human PSCs-derived macrophages differentiation (9, 11). We also screened previously reported basal media suitable for human macrophages induction (10, 28, 45, 46) (Fig. S1, *A*–*C*). We used this serum-free differentiation medium (StemSpan SFEM Ⅱ) with chemically defined conditions to direct pPSC-derived macrophages (pPSCdMs) differentiation (Fig. 1, *A* and *B*). First, porcine EPSCs were differentiated into primitive streak (PS) on day 1 and subsequently into ME on day 2 after the addition of BMP4, activin A, and CHIR99021 (Fig. 1*B*). The most posterior region of PS develops ME progenitor cells and the formation of PS can be defined by the upregulation of *T-Brachyury* (*TBXT*) and *MIXL1* (47, 48). The quantitative reverse transcription PCR (RT-qPCR) results showed that the expression of pluripotency-related genes (such as *POU5F1* (*OCT4*), *SOX2*, and *NANOG*) were gradually downregulated during the formation of ME (Fig. 1*C*). The expression of PS-related genes (such as *MIXL1* and *TBXT*) increased and peaked on day 1 and then decreased on day 2. This result indicated the transient formation of PS (Fig. 1*D*). The expression of ME-related genes (such as *KDR*) was gradually upregulated within 2 days, indicating the formation of ME (Fig. 1*D*). After 2 days of ME induction, more than 94% of cells were KDR^+^ (Fig. 1*H*). From day 2, HE induction with vascular endothelial growth factor A (VEGFA), stem cell factor (SCF), basic fibroblast growth factor (bFGF), and SB431542 was conducted to differentiate ME into HE. HE could be induced on day 5 (Fig. 1*B*), and the expression levels of HE markers including *PECAM1* (*CD31*), *CD34*, *CDH5* (*VE-cadherin*), and *RUNX1* were upregulated (Fig. 1*E*). HE is a specific subtype of cells that expressing *CD34* in endothelial cells and is derived from hematopoietic mesodermal progenitor cells expressing *KDR* (49, 50, 51). After 3 days of HE induction, more than 71% of cells were CD31^+^ (Fig. 1*H*). On day 5, a combination of VEGFA, SCF, bFGF, thrombopoietin, feline McDonough sarcoma-related tyrosine kinase 3 ligand, interleukin 6 (IL6), and interleukin 3 (IL3) was added to induce EMPs(Fig. 1*B*). On day 9, the gene expression levels of EMPs markers, such as *RUNX1*, *CD34*, *CD45*, and *SPI1* (*PU.1*), were upregulated, similar to the HPCs isolated from bone marrow (Fig. 1*F*). After 4 days of EMPs induction, more than 57% of cells were CD45^+^ (Fig. 1*H*). Suspended EMPs were harvested and further differentiated into macrophages with the addition of macrophage colony-stimulating factor 1 for another 2 days. On day 11, mature pPSCdMs could be harvested (Fig. 1*B*) and the expression levels of macrophage-related genes, such as *CD14*, *CD163*, *CSF1R*, *PU.1*, *CSF2RA*, and *CSF2RB*, were significantly upregulated, similar to the PAMs (Fig. 1*G*). After 2 days of macrophage induction, more than 85% of cells were CD163^+^ (Fig. 1*H*). After initially seeding 20,000 pEPSCs on a 24-well culture plate, about 400,000 CD163^+^ macrophages were harvested on day 11, equating to about 20 macrophages generated from one pEPSCs.

To track the fate transitions of pEPSCs during this differentiation process, we analyzed the expression levels of genes characteristic of pluripotency (*POU5F1*, *SOX2*, and *NANOG*), PS (*TBXT* and *MIXL1*), ME (*KDR*), HE (*CD31*, *CD34*, *RUNX1* and *CDH5*), EMPs (*RUNX1*, *CD45*, and *ITGA2B*) and pPSCdMs (*CSF1R*, *CD14*, *CD163*, and *PU.1*) in samples collected throughout the differentiation process (Fig. 1*I*). The expression of the stem cells markers (*POU5F1*, *SOX2*, and *NANOG*) declined immediately, indicating a rapid loss of pluripotency. By contrast, the expression of *TBXT* and *MIXL1* increased and peaked on day 1, indicating the transient formation of PS. The expression of *KDR* increased and that of *TBXT* and *MIXL1* decreased on day 2, indicating the differentiation of PS into mesodermal progenitor cells. The expression of HE markers *CD31*, *CD34*, *CDH5*, and *RUNX1* rapidly increased and peaked on day 5, consistent with the formation of HE. The expression of EMPs markers *RUNX1*, *CD45* and *CD41a* steadily increased on day 5 and day 9, consistent with the formation of EMPs. Finally, the expression of macrophage markers *CD14*, *CD163*, and *PU.1* steadily increased on day 9 and day 11, consistent with the differentiation of macrophages (Fig. 1*I*). Overall, we developed an efficient and rapid protocol that allowed production of EMP-like cells that could be further differentiated toward mature pPSCdMs in 11 days under chemically defined conditions.

### Molecular characterization of pPSCdMs

**Figure 2:**
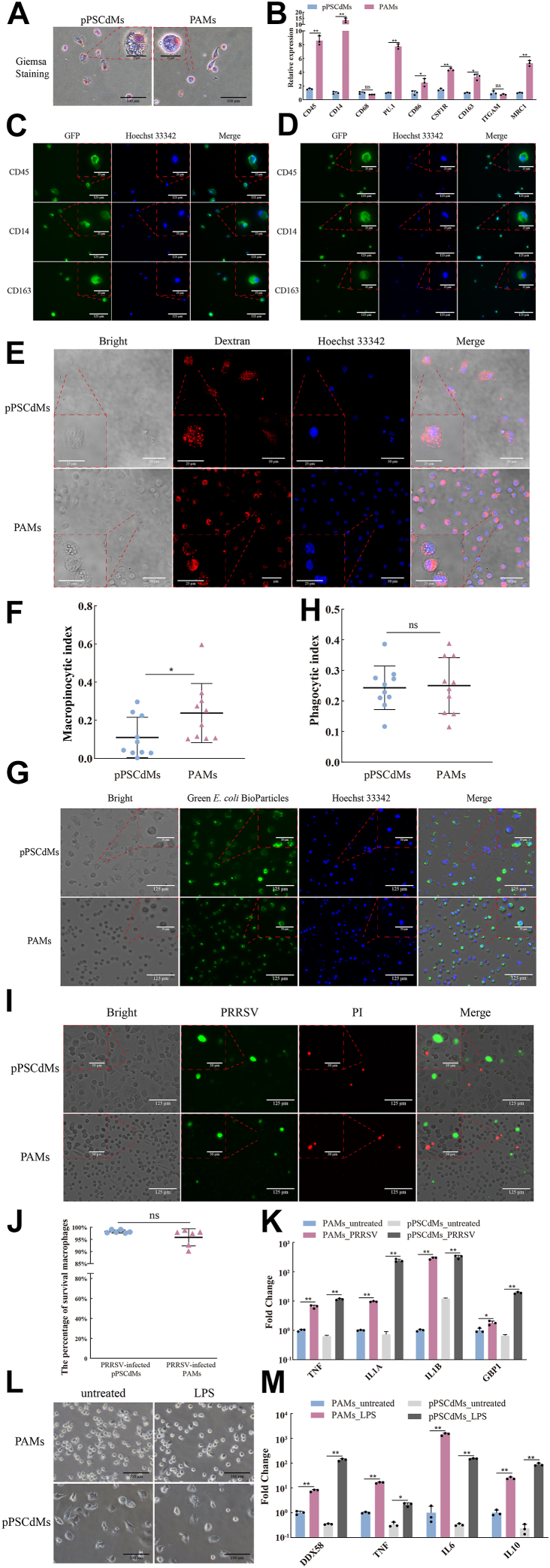
**Characterization of pPSCdMs.***A*, Giemsa staining of pPSCdMs and PAMs. Scale bars represent 25 μm and 100 μm. *B*, the expression levels of macrophage makers in pPSCdMs and PAMs were detected by RT-qPCR. Data indicate mean ± s.d. The *p* values were calculated by using two-tailed *t*-tests. n = 3. *C*, immunofluorescence staining of macrophage markers CD14, CD45, and CD163 in pPSCdMs. Scale bars represent 25 μm and 125 μm. *D*, immunofluorescence staining of macrophage markers CD14, CD45, and CD163 in PAMs. Scale bars represent 25 μm and 125 μm. *E*, representative images of the tetramethylrhodamine-dextran uptake assay in the pPSCdMs and PAMs. Positive dextran-uptake was shown in *red*; cell nuclei were stained with Hoechst 33342 in *blue*. Scale bars represent 25 μm and 50 μm. Unless taken up by endocytic process, dextran conjugates that carried a *red-orange* fluorescence were membrane impermeant. Macrophages would have red fluorescent puncta after taking up dextran. *F*, the macropinocytic index of pPSCdMs and PAMs. The macropinocytic index was the percentage of dextran-positive cells area accounting for the total cells area during the same dextran-uptake time and the macropinocytic index of PAMs was significantly higher than pPSCdMs. Data indicate mean ± s.d. The *p* values were calculated by using two-tailed *t*-tests. n = 9. *G*, representative images of the phagocytosis of pPSCdMs and PAMs to Green *E. coli* BioParticles. Positive *E. coli* BioParticles uptake were shown in *green*; cell nuclei were stained with Hoechst 33342 in *blue*. Scale bars represent 50 μm and 125 μm. *H*, the phagocytic index of pPSCdMs and PAMs. The phagocytic index was the percentage of positive macrophages multiplied by their mean of fluorescence and was normalized on 20 μM cytochalasin-treated samples. There was no significant difference in phagocytosis between pPSCdMs and PAMs. Data indicate mean ± s.d. The *p* values were calculated by using two-tailed *t*-tests. n = 9. *I*, composite bright-field and fluorescent images of pPSCdMs and PAMs after PRRSV infection for 12 h, stained for PRRSV (*green*) and propidium iodide (PI) (*red*). The dead cell nuclei were stained with PI and presented *red fluorescence*. Scale bars represent 50 μm and 125 μm. *J*, the net survival rate for pPSCdMs and PAMs after PRRSV infection for 12 h. The net survival rate was the proportion of survival cells in total cells. Data indicate mean ± s.d. The *p* values were calculated by using two-tailed *t*-tests. n = 6. *K*, the expression levels of *TNF*, *IL1A*, *IL1B*, and *GBP1* in pPSCdMs and PAMs after PRRSV infection for 12 h. Data indicate mean ± s.d. The *p* values were calculated by using two-tailed *t*-tests. n = 3. *L*, the bright-field images of pPSCdMs and PAMs after LPS stimulation for 24 h. Scale bars represent 100 μm. *M*, the expression levels of *DDX58*, *TNF*, *IL6*, and *IL10* in pPSCdMs and PAMs after LPS stimulation for 24 h. Data indicate mean ± s.d. The *p* values were calculated by using two-tailed *t*-tests. n = 3. GBP1, guanylate-binding protein 1; IL, interleukin; LPS, lipopolysaccharide; PAMs, porcine alveolar macrophages; pPSCdMs, pPSC-derived macrophages; PRRSV, porcine respiratory and reproductive syndrome virus; RT-qPCR, quantitative reverse transcription PCR; TNF, tumor necrosis factor.

We then identified pPSCdMs by morphology and cell surface markers. pPSCdMs showed typical macrophage morphology (Fig. 2*A*). Compared with PAMs, they were larger in size, with the cytoplasm containing vacuoles, similar to the human PSC-derived macrophages (9). Compared with PAMs, the pPSCdMs also expressed the classical markers of macrophages, such as *CD45*, *CD14*, *CD68*, *PU.1*, *CD86*, *CSF1R*, *CD163*, *ITGAM* (*CD11b*), and *MRC1* (*CD206*; Fig. 2*B*). Macrophages markers were highly expressed in both PAMs and pPSCdMs, although some genes in pPSCdMs were lower in PAMs. Immunofluorescence staining also showed that pPSCdMs, similar to PAMs, expressed the macrophage markers, such as CD45, CD14, and CD163 (Fig. 2, *C* and *D*). Overall, the pPSCdMs exhibited typical macrophage morphology and macrophage markers, similar to PAMs.

### Functional characterization of pPSCdMs

**Supporting Figure S2:**
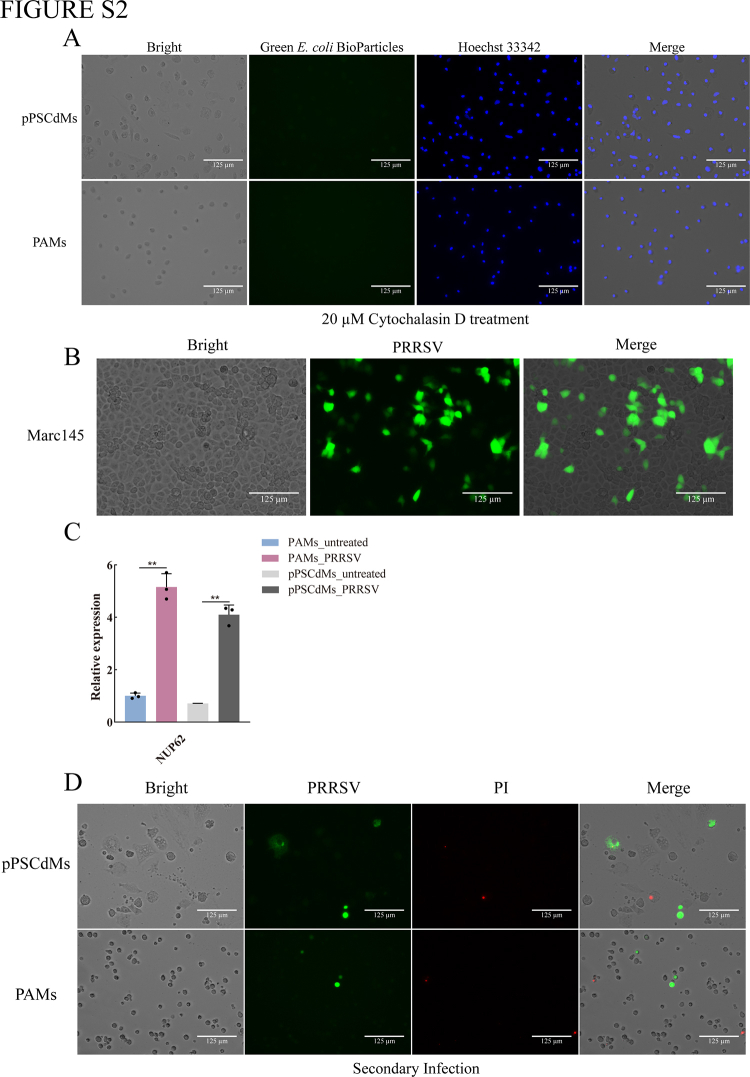
*A*, representative images of the phagocytosis of pPSCdMs and PAMs treated with 20 μM Cytochalasin D to Green *E. coli* BioParticles. Positive uptake *E. coli* BioParticles were shown in *green*; cell nuclei were stained with Hoechst 33342 in *blue*. Scale bars represent 125 μm. *B*, composite bright-field and fluorescent image of Marc145 after PRRSV infection for 12  h, stained for PRRSV (*green*). Scale bars represent 125 μm. *C*, the expression level of *NUP62* in pPSCdMs and PAMs after PRRSV infection for 12 h was detected by RT-qPCR. Data indicate mean ± s.d. The *p* values were calculated by using two-tailed *t*-tests. n = 3. *D*, the supernatant cultures of pPSCdMs and PAMs at 72 h after PRRSV infection were collected for secondary infection. Composite bright-field and fluorescent image of secondary PRRSV-infected pPSCdMs and PAMs for 12  h, stained for PRRSV (*green*) and PI (*red*). Scale bars represent 125 μm.

To further evaluate the function of pPSCdMs, we first compared the endocytosis of pPSCdMs and PAMs *via* tetramethylrhodamine dextran according to a previously reported experimental procedure (52). Both pPSCdMs and PAMs had the ability to ingest dextran, and they showed red fluorescent puncta inside each macrophage (Fig. 2*E*). The macropinocytic index of PAMs was significantly higher than that of pPSCdMs (Fig. 2*F*). We then compared the phagocytosis of pPSCdMs and PAMs to pathogen by using Green *Escherichia coli* BioParticles. Both pPSCdMs and PAMs demonstrated phagocytic ability for pathogens, and green fluorescence inside macrophages were observed (Figs. 2*G* and S2*A*). No significant difference in phagocytosis was observed between pPSCdMs and PAMs (Fig. 2*H*). These results indicated that pPSCdMs demonstrated the ability of endocytosis and phagocytosis for pathogens, similar to PAMs.

We also compared the ability of pPSCdMs to infect PRRSV. GFP-labeled PRRSV (GFP-PRRSV) was incubated with pPSCdMs, PAMs, and Marc145 for 12 h, and they were all infected efficiently by GFP-PRRSV (Figs. 2*I* and S2*B*). Propidium iodide (PI) staining showed that the net survival rate for pPSCdMs and PAMs after infection with PRRSV for 12 h was over 90%, indicating that most pPSCdMs and PAMs could survive in the early stages of infection (Fig. 2, *I* and *J*). Consistent with previous reports (53), the expression of related genes, such as *IL1A*, *IL1B*, tumor necrosis factor (*TNF*), and guanylate-binding protein 1 (*GBP1*), was upregulated in PRRSV-infected pPSCdMs, similar to the response of PAMs (Fig. 2*K*). These results indicated that pPSCdMs demonstrated the ability of PRRSV infection, similar to PAMs. Due to cytopathy, most pPSCdMs and PAMs were dead at 48 to 72 h after PRRSV infection. Subsequently, we demonstrated that pPSCdMs supported PRRSV replication. The expression of *NUP62*, which inhibited host antiviral protein expression (54), was significantly upregulated in PRRSV-infected pPSCdMs, similar to PAMs (Fig. S2*C*). The supernatant cultures of pPSCdMs and PAMs at 72 h after PRRSV infection were collected for secondary infection. Both pPSCdMs and PAMs could be secondary infected by their supernatant cultures (Fig. S2*D*). These results indicated that PRRSV could replicate in pPSCdMs, similar to PAMs. In conclusion, the pPSCdMs could serve as effective hosts for PRRSV infection and replication, similar to PAMs.

Finally, we assessed the response of pPSCdMs and PAMs to LPS stimulation. Consistent with previous reports (42, 55), the innate immune response gene *DDX58* (*RIG-1*), proinflammatory cytokines *IL6* and *TNF*, and anti-inflammatory cytokine *IL10* were upregulated in pPSCdMs treated with LPS for 24 h. This result was also similar to the response of PAMs (Fig. 2, *L* and *M*). In summary, the pPSCdMs exhibited the typical function of macrophages, similar to PAMs, and could provide the platforms of pathogen–host interaction mechanisms.

### RNA-seq of differentiation from pEPSCs to pPSCdMs

**Figure 3:**
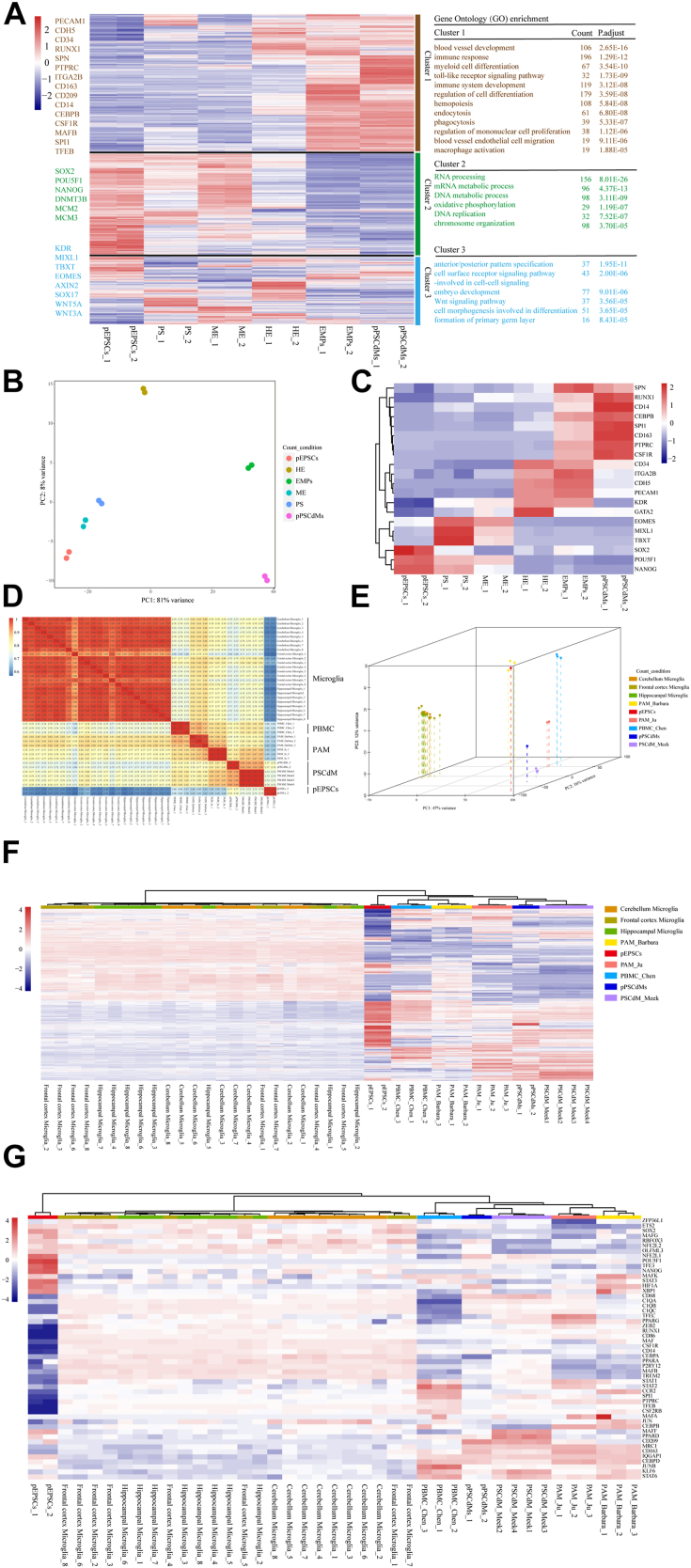
**The RNA-seq of differentiation from pEPSCs to pPSCdMs.***A*, heatmap illustrating the key differences linked to the differentiation of macrophages from pEPSCs. Represented genes of each cluster were listed on the *left*. Gene ontology enrichments for each cluster were presented on the *right*. −2∼2 indicates scaled expression. *B*, PCA analysis of RNA-seq replicates for all differentiation stage of pEPSCs, PS, ME, HE, EMPs, and pPSCdMs. *C*, heatmap showing the expression of genes characteristic of pluripotency (*POU5F1*, *SOX2*, and *NANOG*), PS (*TBXT* and *MIXL1*), ME (*EOMES* and *KDR*), HE (*CD31*, *CD34*, *CDH5*, and *GATA2*), EMPs (*RUNX1*, *SPN*, *CD45*, *ITGA2B*, and *PU.1*) and macrophages (*CSF1R*, *CD14*, *CD163*, and *CEBPB*). −2∼2 indicates scaled expression. *D*, Pearson correlation coefficient for microglia, PBMC_Chen, pEPSCs, pPSCdMs, PSCdM_Meek, PAM_Ju, and the PAMs_Barbara based on RNA-seq. The color depth indicated the correlation coefficient between samples. The closer the color was to *red* (the closer the correlation coefficient was to 1), the greater the correlation; the closer it was to *blue*, the less relevant. *E*, PCA for microglia, PBMC_Chen, pEPSCs, pPSCdMs, PSCdM_Meek, PAM_Ju, and PAMs_Barbara based on RNA-seq. *F*, hierarchical Clustering analysis of 100 highly expressed genes among microglia, PBMC_Chen, pEPSCs, pPSCdMs, PSCdM_Meek, PAM_Ju, and PAMs_Barbara based on RNA-seq. −4∼4 indicates scaled expression. *G*, heatmap showing the expression of macrophage-related genes among microglia, PBMC_Chen, pEPSCs, pPSCdMs, PSCdM_Meek, PAM_Ju, and PAMs_Barbara based on RNA-seq. −4∼4 indicates scaled expression. EMPs, erythromyeloid progenitor cells; HE, hematopoietic endothelium; ME, mesoderm; PAMs, porcine alveolar macrophages; PBMCs, peripheral blood mononuclear-derived macrophages; PCA, principal component analysis; pEPSCs, porcine expanded potential stem cells; pPSCdMs, pPSC-derived macrophages; PS, primitive streak.

To further gain insights into the gene expression dynamics of the macrophage differentiation, we performed total RNA-seq on pEPSCs, PS, ME, HE, EMPs, and pPSCdMs. Then, we aggregated the differentially expressed genes (DEGs) and divided them into three clusters. Gene Ontology (GO) enrichment analysis was performed on DEGs in each cluster. GO analysis of DEGs in cluster 2 revealed significant enrichment in GO terms “RNA processing,” “mRNA metabolic process,” “DNA metabolic process,” “oxidative phosphorylation,” “DNA replication,” and “chromosome organization.” The genes in cluster 2, largely involved in the regulation of pluripotency (*e.g.*, *POU5F1*, *SOX2*, and *NANOG*) and DNA replication (*e.g.*, *MCM2* and *MCM3*), were gradually downregulated (Fig. 3*A* cluster 3). These results indicated the rapid decline of pluripotency and the loss of proliferation ability during macrophages differentiation.

GO analysis of DEGs in cluster 3 revealed significant enrichment in GO terms “anterior/posterior pattern specification,” “cell surface receptor signaling pathway-involved in cell-cell signaling,” “embryo development,” “Wnt signaling pathway,” “cell morphogenesis involved in differentiation,” and “formation of primary germ layer.” The genes in cluster 3 (*e.g.*, *TBXT*, *MIXL1*, *EOMES*, *AXIN2*, and *KDR*) were transiently upregulated and then downregulated along differentiation (Fig. 3*A* cluster 2). The Wnt signaling pathway plays a vital role in the formation of PS and ME (56, 57). It represents that pEPSCs transitioned toward hematopoietic fate after a brief differentiation of hematopoietic mesodermal progenitor cells.

GO analysis of DEGs in cluster 1 revealed significant enrichment in GO terms “regulation of cell differentiation,” “blood vessel development,” “blood vessel endothelial cell migration,” “hemopoiesis,” “myeloid cell differentiation,” “regulation of mononuclear cell proliferation,” “immune system development,” “macrophage activation,” “immune response,” “toll-like receptor signaling pathway,” “endocytosis,” and “phagocytosis.” The genes in cluster 1 (*e.g.*, *PECAM1*, *CDH5*, *CD34*, and *RUNX1*), enriched in the “regulation of cell differentiation,” “blood vessel development,” “blood vessel endothelial cell migration,” were upregulated at HE stage and downregulated at EMPs stage along differentiation. Lastly, the genes in cluster 1 (*e.g.*, *SPN*, *PTPRC*, *ITGA2B*, *CD163*, *CD209*, *CD14*, *CEBPB*, *CSF1R*, *MAFB*, *SPI1*, and *TFEB*), enriched in “hemopoiesis,” “myeloid cell differentiation,” “regulation of mononuclear cell proliferation,” and “immune system development,” were upregulated at EMPs and pPSCdMs stage along differentiation, suggesting the formation of myeloid cells (Fig. 3*A* cluster 1). Principal component analysis (PCA) of RNA-seq datasets showed that the biological replicates were clustered together and the clusters of pEPSCs, PS, and ME groups were adjacent. The clusters of HE, EMPs, and pPSCdMs groups were scattered (Fig. 3*B*). Heatmap also showed the expression of pluripotency-related genes (*POU5F1*, *SOX2*, and *NANOG*), PS-related genes (*TBXT* and *MIXL1*), ME-related gene (*EOMES* and *KDR*), HE-related genes (*CD31*, *CD34*, *CDH5*, *GATA2*, and *RUNX1*), EMP-related genes (*RUNX1*, *SPN*, *CD45*, *ITGA2B*, and *PU.1*), and macrophage-related genes (*CSF1R*, *CD14*, *CD163*, and *CEBPB*) based on RNA-seq datasets (Fig. 3*C*).

In addition, we compared the transcriptional features of pPSCdMs in this manuscript with the PSCdM from EBs (PSCdM_Meek) (42), microglia (58), peripheral blood mononuclear derived macrophages (PBMCs_Chen) and porcine alveolar macrophages (PAMs_Barbara and PAMs_Ju) (42, 59). Pearson correlation coefficient and PCA showed that the pPSCdMs was very close to the PSCdM_Meek (Fig. 3, *D* and *E*). The transcriptional features of pPSCs-derived macrophages were very similar, although the differentiation methods varied. Tissue-resident macrophages, such as PAMs, microglia, and Kupffer cells, are derived from EMPs generated in the second hematopoiesis (19, 23, 24, 25). PBMCs and BMDMs are derived from HPCs generated in the third hematopoiesis (29, 30, 31, 32). Pearson correlation coefficient and PCA also showed that pPSC-derived macrophages from different differentiation methods were similar to PAMs and PBMCs but different from microglia (Fig. 3, *D* and *E*). According to previous reports, the differentiation methods of pPSCdMs and PAMs were similar, and they underwent myeloid differentiation independent of the *MYB* gene. Therefore, we believe that pPSC-derived macrophages *in vitro* could effectively mimic isolated tissue-resident macrophages *in vivo*. Hierarchical clustering analysis of 100 highly expressed genes among pEPSCs, PBMCs, pPSCdMs, PSCdM_Meek, PAMs_Barbara and PAMs_Ju also supported this conclusion (Fig. 3*F*). Heatmaps also showed the expression of macrophage marker genes between different macrophages (Fig. 3*G*). The classical markers of macrophages, such as *CD45*, *CD14*, *CD68*, *PU.1*, *CD86*, *CSF1R*, *CD163*, *ITGAM*, and *MRC1* (Fig. 3*G*), were highly expressed in pPSCdMs, PSCdM_Meek, and PAMs, consistent with the previous results of RT-qPCR (Fig. 2*B*).

In summary, the transcriptional features of pPSCs-derived macrophages from different differentiation methods were highly similar, and pPSCs-derived macrophages could successfully mimic isolated tissue-resident macrophages.

### Activation of the TGF-β signaling pathway was required in the formation of mesoderm

**Figure 4:**
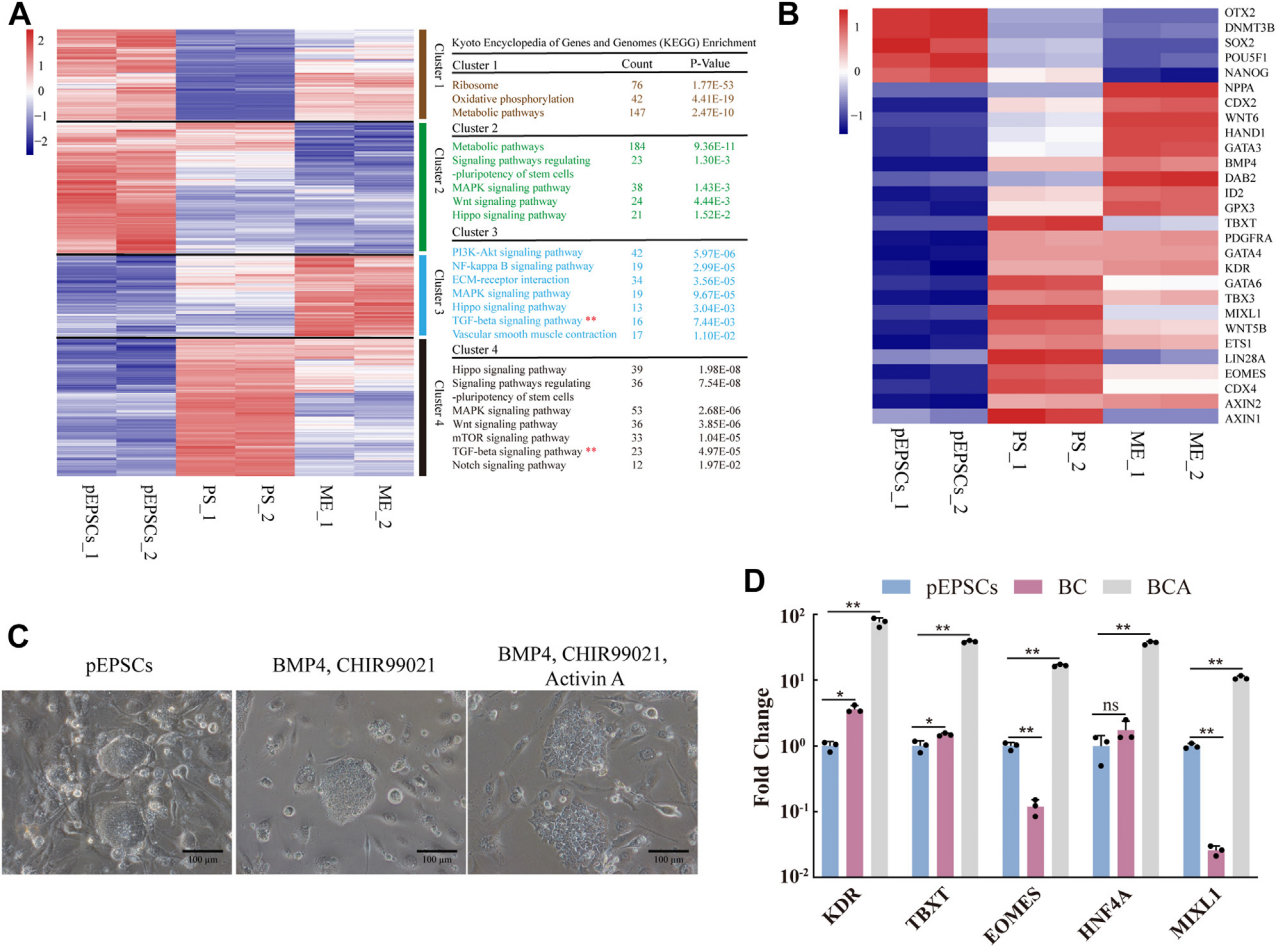
**The activation of TGF-β signaling pathway promoted the formation of mesoderm.***A*, four clusters of DEGs highlighted the key differences during the differentiation of mesoderm from pEPSCs. Represented genes of each cluster were listed on the *left*. KEGG enrichments for each cluster were presented on the *right*. −2∼2 indicates scaled expression. *B*, heatmap showing the expression of genes characteristic of pluripotency (*POU5F1*, *SOX2*, and *NANOG*), PS (*TBXT* and *MIXL1*), ME (*EOMES* and *KDR*). −1∼1 indicates scaled expression. *C*, the bright-field images of cells differentiated from pEPSCs with the addition of BMP4, CHIR99021 (BC) and BMP4, CHIR99021, activin A (BCA). Scale bars represent 100 μm. *D*, the expression levels of mesodermal genes between the BC group and BCA group were detected by RT-qPCR. Data indicate mean ± s.d. The *p* values were calculated by using two-tailed *t*-tests. n = 3. BC, BMP4, CHIR99021; BCA, BMP4, CHIR99021, and activin A; BMP4, basic morphogenetic protein 4; DEGs, differentially expressed genes; KEGG, Kyoto Encyclopedia of Genes and Genomes; ME, mesoderm; pEPSCs, porcine expanded potential stem cells; PS, primitive streak; RT-qPCR, quantitative reverse transcription PCR; TGF-β, transforming growth factor beta.

As previously reported, the TGF-β signaling pathway plays an important role in the formation of ME. First, we explored the effect of TGF-β signaling on hematopoietic ME differentiation. We aggregated the DEGs and divided them into four clusters during ME differentiation. Kyoto Encyclopedia of Genes and Genomes (KEGG) enrichment analysis was performed on DEGs in each cluster. KEGG analysis of DEGs in cluster 1 and cluster 2 revealed significant enrichment in “oxidative phosphorylation,” “metabolic pathways,” and “signaling pathways regulating pluripotency of stem cells” (Fig. 4*A*), and the expression of pluripotency-related genes (such as *POU5F1*, *SOX2*, and *NANOG*) was gradually downregulated during differentiation (Fig. 4*B*). These results indicated the loss of pluripotency and the beginning of the differentiation process. KEGG analysis of DEGs in cluster 3 and cluster 4 revealed significant enrichment in “PI3K-Akt signaling pathway,” “Hippo signaling pathway,” “signaling pathways regulating pluripotency of stem cells,” “MAPK signaling pathway,” “Wnt signaling pathway,” “TGF-beta signaling pathway” (Fig. 4*A*). The expression of genes (such as *EOMES*, *TBXT*, *GATA6*, *LIN28A*, *CDX4*, *AXIN1*, *WNT5B*, and *MIXL1*) was upregulated at PS stage and downregulated at ME stage, indicating the transient formation of PS. The expression levels of genes (such as *GATA3*, *GATA4*, *BMP4*, *CDX2*, *PDGFRA*, *KDR*, *WNT6*, *AXIN2*, and *ETS1*) were gradually upregulated in the formation of ME (Fig. 4*B*). According to the results of RNA-seq analysis, during the ME differentiation of pEPSCs, the TGF-β signaling pathway was enriched in the upregulated genes in PS and ME (Fig. 4*A*). Whether the TGF-β signaling plays a vital role in ME formation remains unclear. Furthermore, we investigated the role of the TGF-β signaling in the formation of porcine ME, and pEPSCs could be differentiated into ME with the addition of BMP4, CHIR99021, and activin A. However, the formation of hematopoietic ME was blocked from PSCs with the addition of BMP4 or CHIR99021 alone. The induced cells maintained the classical clonal morphology with the addition of BMP4 or CHIR99021 alone (Fig. 4*C*). The RT-qPCR results showed that the ME marker genes (*MIXL1* and *EOMES*) and endoderm genes (*HNF4A*) had no significant upregulation (Fig. 4*D*). With the addition of BMP4, CHIR99021, and activin A, the induced cells showed differentiation morphology (Fig. 4*C*), and the mesodermal marker genes (*TBXT*, *MIXL1*, *EOMES*, and *KDR*) and endoderm genes (*HNF4A*) were significantly upregulated (Fig. 4*D*). Therefore, the formation of ME requires the activation of the TGF-β signaling pathway with the addition of activin A, which effectively promoted the formation of ME.

### Inhibition of the TGF-β signaling pathway enhanced the fate transformation of hematopoiesis

**Figure 5:**
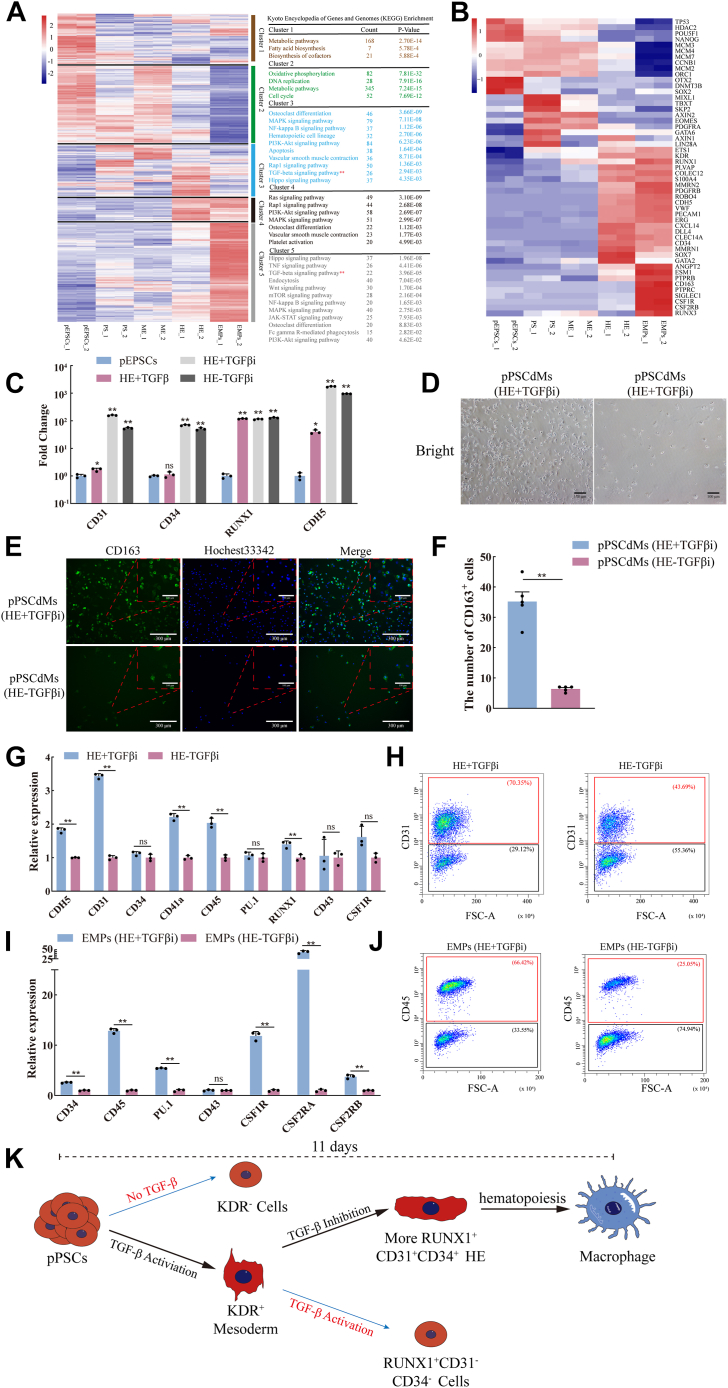
**The inhibition of TGF-β signaling pathway enhanced the fate transformation of hematopoiesis.***A*, five clusters of DEGs highlighted the key differences during the differentiation of EMPs from porcine EPSCs. Represented genes of each cluster were listed on the *left*. KEGG enrichments for each cluster were presented on the *right*. −2∼2 indicates scaled expression. *B*, heatmap showing the expression of genes characteristic of pluripotency (*POU5F1*, *SOX2*, and *NANOG*), PS (*TBXT* and *MIXL1*), ME (*EOMES* and *KDR*), HE (*CD31*, *CD34*, *CDH5*, and *GATA2*) and EMPs (*RUNX1*, *SPN*, *CD45*, *ITGA2B*, and *PU.1*). −1∼1 indicates scaled expression. *C*, the expression levels of HE genes in pEPSCs, HE+TGFβ, HE-TGFβi, and HE+TGFβi groups were detected by RT-qPCR. Data indicate mean ± s.d. The *p* values were calculated by using two-tailed *t*-tests. n = 3. *D*, the bright-field images of pPSCdMs differentiated from HE-TGFβi group and HE+TGFβi group. Scale bars represent 100 μm. *E*, immunofluorescence staining of the CD163^+^ cells differentiated from HE-TGFβi group and HE+TGFβi group. Scale bars represent 100 μm and 300 μm. *F*, the number of the CD163^+^ cells differentiated from HE-TGFβi group and HE+TGFβi group. The *p* values were calculated by using two-tailed *t*-tests. n = 5. *G*, the expression levels of HE genes between HE-TGFβi group and HE+TGFβi group were detected by RT-qPCR. Data indicate mean ± s.d. The *p* values were calculated by using two-tailed *t*-tests. n = 3. *H*, FACS analysis of CD31^+^ cells between HE-TGFβi group and HE+TGFβi group. The percentages of positive populations are shown in *red*. *I*, the expression levels of EMPs genes between EMPs (HE-TGFβi) and EMPs (HE+TGFβi) groups were detected by RT-qPCR. Data indicate mean ± s.d. The *p* values were calculated by using two-tailed *t*-tests. n = 3. *J*, FACS analysis of CD45^+^ cells between EMPs (HE-TGFβi) group and EMPs (HE+TGFβi) group. The percentages of positive populations are shown in *red*. *K*, schematic overview of the function of TGF-β signaling pathway in the fate transformation of hematopoiesis. DEGs, differentially expressed genes; EMPs, erythromyeloid progenitor cells; EPSCs, expanded potential stem cells; FACS, fluorescence-activated cell sorting; HE, hematopoietic endothelium; KEGG, Kyoto Encyclopedia of Genes and Genomes; ME, mesoderm; pEPSCs, porcine expanded potential stem cells; pPSCdMs, pPSC-derived macrophages; PSCs, pluripotent stem cells; RT-qPCR, quantitative reverse transcription PCR; TGF-β, transforming growth factor beta.

Through the RNA-seq analysis, we aggregated the DEGs and divided them into five clusters during EMPs differentiation. KEGG enrichment analysis was performed on DEGs in each cluster. KEGG analysis of DEGs in cluster 1 and cluster 2 revealed significant enrichment in “metabolic pathways,” “oxidative phosphorylation,” “DNA replication,” and “cell cycle” (Fig. 5*A*). The expression of the *ORC1*, *POU5F1*, *NANOG*, and *MCM* family genes was downregulated in hematopoiesis (Fig. 5*B*), indicating that the cell proliferation ability gradually decreased during the hematopoietic process. KEGG analysis of DEGs in cluster 3, cluster 4, and cluster 5 revealed significant enrichment in “hematopoietic cell lineage,” “vascular smooth muscle contraction,” “TGF-beta signaling pathway,” “Hippo signaling pathway,” and “osteoclast differentiation” (Fig. 5*A*), indicating the progress of the hematopoiesis. The expression of hematopoiesis related genes, such as *CD34*, *RUNX1*, *RUNX3*, *ESM1*, *PTPRB*, *PTPRC*, and *GATA2*, gradually increased during the hematopoietic process (Fig. 5*B*).

The activation of the TGF-β signaling pathway is required in the formation of ME. We then determined the role it plays in the formation of HE. Subsequently, we explored the effect of TGF-β signaling in hematopoiesis. During hematopoiesis, the TGF-β signaling pathway was enriched at the stages of HE and EMPs (Fig. 5*A*). On the basis of the addition of VEGFA, FGF2, and SCF, CD31^+^CD34^+^RUNX1^+^ HE would be induced whether the TGF-β inhibitor (SB431542) was added or not. The RT-qPCR results showed that the expression levels of *CD31*, *CD34*, *CDH5* and *RUNX1* were upregulated in both HE-TGFβi and HE+TGFβi groups. However, with the addition of TGF-β activator (activin A), the ME could not be induced into HE, and the induced cells were CD31^-^CD34^-^RUNX1^+^ cells. The RT-qPCR results showed that only *CDH5* and *RUNX1* were upregulated and the expression of *CD31* and *CD34* had no significant change in the HE+TGFβ group (Fig. 5*C*). Subsequently, we further induced HE into macrophages, and we found that macrophages could be induced regardless of whether TGF-β inhibitors (SB431542) were added at the HE stage. However, a higher number of macrophages could be harvested after the addition of TGF-β inhibitors at the HE stage compared with the absence of TGF-β inhibitor (Fig. 5, *D*–*F*). We found that HE could be induced regardless of whether TGF-β inhibitors were added at the HE stage. Moreover, more CD31^+^CDH5^+^ HE were induced significantly after TGF-β inhibitor was added at the HE stage compared with no TGF-β inhibitor. When the TGF-β inhibitor was not added at the HE stage, the proportion of CD31^+^ cells was 43%. When TGF-β inhibitors were added at the HE stage, the proportion of CD31^+^ cells was 70% (Fig. 5, *G* and *H*). Then, HE cells were further differentiated into the EMPs. When TGF-β inhibitors were not added at the HE stage, the proportion of CD45^+^ cells was 25%. When TGF-β inhibitors were added at the HE stage, the proportion of CD45^+^ cells was 66% (Fig. 5, *I* and *J*). These phenomena indicated that the activation of the TGF-β signaling pathway is required in the formation of ME. Although the inhibition of TGF-β signaling pathway did not affect the formation of HE, it will affect the induction efficiency of ME to HE, and the inhibition of the TGF-β signaling pathway promoted the fate transformation of hematopoiesis (Fig. 5*K*).

## Discussion

Given the high similarity of physiology, anatomy, and metabolism between humans and pigs, pigs are considered the best donor for human organ xenotransplantation and a good model for cultivating humanized organs (60, 61, 62). However, immune rejection has limited its further application. As one of the important immune cells in organism, macrophages play an important role in immune rejection. Establishing the differentiation model of porcine macrophages *in vitro* and further analyzing the characteristics of porcine macrophages will lay a good foundation for the comprehensive analysis of the differences between human and porcine macrophages. Results of this work are of great significance to reduce immune rejection, cultivate humanized organs, and xenotransplantation in the future. As the limitations of primary macrophages isolated *in vivo*, macrophages derived from PSCs can replace primary macrophages, providing a broad prospect for the platforms of pathogen–host interaction mechanisms and the analysis of macrophage features. Here, we firstly established an efficient and rapid induction differentiation system from pEPSCs. We demonstrated that the pPSCdMs showed typical macrophage morphology, and they expressed macrophages surface markers and specific transcription factors of macrophages. Moreover, they had the function of macrophages similar to PAMs. These findings laid a valuable foundation for the establishment of the platforms of host–pathogen interaction mechanisms and the comprehensive analysis of the features between human and porcine macrophages in the future.

In terms of cytokines, the differentiation protocol in this manuscript mainly utilized the human macrophages differentiation protocols. However, we found that basal medium also played an important role in the differentiation of macrophages, which was also consistent with previous reports (28). Moreover, we found that these basal media used for human macrophages differentiation (10, 28, 45, 46) were not suitable for porcine macrophages differentiation with the addition of same cytokines (Fig. S1, *A*–*C*). After screening different basal media with the addition of same cytokines, we found that only the third group of basal medium (StemSpan SFEM Ⅱ) could be used for porcine macrophages differentiation, which could differentiate pEPSCs into CD45^+^ EMPs. The TGF-β signaling pathway plays an important role in the hematopoietic development. Here, we explored its complex function during porcine hematopoietic development. The TGF-β signaling pathway plays an active role in the formation of porcine ME, which requires the activation of the TGF-β signaling pathway with the addition of activin A. The absence of the TGF-β signaling pathway during ME formation did not differentiate pEPSCs into ME (Fig. 4, *C* and *D*). However, the activation of the TGF-β signaling pathway played a negative role in the formation of HE. The activation of the TGF-β signaling pathway blocked the formation of CD31^+^CD34^+^RUNX1^+^ HE (Fig. 5*C*). More CD31^+^ HE and CD45^+^ EMPs could be harvested only when the TGF-β signaling pathway was inhibited in the HE phase (Fig. 5, *G*–*K*).

The RNA-seq results revealed that the transcriptional profiles of pPSCdMs overlapped with those of PSCdM_Week (42); although all of them were different from PAMs (Fig. 3, *D* and *E*). Some differences were observed between PSC-derived macrophages and tissue-resident macrophages, and the transformation of PSC-derived macrophages to PAMs must be further induced. Human and mouse BMDMs could be further specialized into alveolar macrophages (63, 64). The transition from PSCs-derived macrophages to PAMs needs to be further explored. Despite being differentiated from pEPSCs *in vitro*, pPSCdMs also showed many key functions similar to those of macrophages isolated *in vivo*. The pPSCdMs have the functional abilities for endocytosis, phagocytosis, and the response to LPS stimulation. Notably, pPSCdMs also served as targets for infection by major pig pathogens, including PRRSV. Thus, pPSCdMs can serve as a platform for studying host–pathogen interaction mechanisms. On the basis of the results of RNA-seq and RT-qPCR, a small number of EMPs began to appear at the late stage of HE. This phenomenon could be the reason for the upregulation of *CD41a*, *CD45*, and *PU.1* at the HE stage (Figs. 1*E* and 3*C*). Moreover, a small number of macrophages began to appear at the EMPs stage. The upregulation of macrophage marker genes, such as *CD14* and *CD163*, could be detected at the stage of EMPs (Fig. 5*B*). Thus, our induction system was highly suitable for the differentiation of macrophages; moreover, the differentiation time of macrophages was shortened, and pPSCdMs could be obtained on day 11. These results strongly supported that our induction system was efficient, rapid, and safe, which provided a valuable platform for screening of new drugs and genes, host–pathogen interaction mechanisms, and xenotransplantation.

In recent years, the single-cell RNA sequencing (scRNA-seq) has revealed the expression of all genes in the whole genome from single-cell resolution, which has promoted the study of highly heterogeneous cell populations. The high-throughput sequencing technology represented by scRNA-seq can easily and quickly establish the tissue development pedigree of biological individuals (22, 26, 65). In the future, we will further explore the differentiation process of porcine macrophages by using single-cell sequencing technology.

## Experimental procedures

### Culturing mouse STO feeder cells

STO feeder cells were passaged every 3 days and cultured at 37 °C/5% CO_2_, under normoxia conditions, in STO medium. The STO medium contains Dulbecco's modified Eagle's medium (DMEM) (CORNING), 10% fetal bovine serum (FBS) (Vistech, New Zealand), 1% penicillin/streptomycin (P/S) (Thermo Fisher Scientific, cat. no. 15140122), 2 mM GlutaMAX (Thermo Fisher Scientific, cat. no. 35050061), 1% NEAA (Thermo Fisher Scientific, cat. no. 11140050), 0.1% β-mercaptoethanol (Thermo Fisher Scientific, cat. no. 21985023).

The feeder layer was prepared by treating mouse STO feeder cells with mitomycin for 12 h. STO feeder cells were passaged by washing twice with PBS then incubating with 0.05% trypsin/EDTA (Thermo Fisher Scientific, cat. no. 25300062) for 5 min at 37 °C/5% CO_2_. STO feeder cells were centrifuged at 300*g* for 5 min with the STO medium. After removing the supernatant, STO feeder cells were resuspended and plated on 0.1% gelatinized 35 mm-tissue culture plastic at a density of 8 × 10^5^ cells. The STO was free from *mycoplasma* and was detected by PCR.

### Culturing porcine EPSCs

Porcine EPSCs (pEPSCs) were cultured on mitotically-inactivated STO feeder layers in pEPSCs medium as previous reports (44). The pEPSCs not only expressed pluripotent genes (such as *OCT4*, *NANOG*, and *SOX2*) and had the ability of three germ layers differentiation, but also had the ability of trophoblast differentiation. The pEPSCs medium contains KO DMEM (Thermo Fisher Scientific, cat. no. 10829018), 0.3% FBS (Vistech, New Zealand), 0.5% N2 supplement (Thermo Fisher Scientific, cat. no. 17502048), 1% B27 supplement (Thermo Fisher Scientific, cat. no. 17502048), 1% penicillin/streptomycin (P/S) (Thermo Fisher Scientific, cat. no. 15140122), 2 mM GlutaMAX (Thermo Fisher Scientific, cat. no. 35050061), 1% NEAA (Thermo Fisher Scientific, cat. no. 11140050), 0.1% β-mercaptoethanol (Thermo Fisher Scientific, cat. no. 21985023), 65 μg/ml vitamin C (Sigma-Aldrich, cat. no. 49752), 2.5 μM XAV939 (Sigma-Aldrich, cat. no. X3004), 0.2 μM CHIR99021 (Toris, cat. no. 4423), 0.15 μM WH-4-023 (Toris, cat. no. 5413), 20 ng/ml activin A (PeproTech, cat. no. 120-14E), 10 ng/ml LIF (PeproTech, cat. no. 300-05). Porcine EPSCs were passaged every 2 to 3 days by washing twice with PBS then incubating with 0.05% trypsin/EDTA (Thermo Fisher Scientific, cat. no. 25300062) for 5 min at 37 °C/5% CO_2_. Porcine EPSCs were centrifuged at 300*g* for 5 min with the addition of DMEM (10% FBS). After removing the supernatant, porcine EPSCs were resuspended and cultured in pEPSCs medium supplemented with 5% FBS (Vistech, New Zealand) and 5 μM Y27632 (Selleck, cat. no. S1049). The pEPSCs was free from *mycoplasma* and was detected by PCR.

### Culturing PAMs and Marc145

PAMs were obtained from Chengbao Wang’ lab (66), and PAMs were cultured at 37 °C/5% CO_2_, under normoxia conditions, in PAMs medium. The PAMs medium contains RPMI-1640 (CORNING), 10% FBS (Vistech, New Zealand), 1% penicillin/streptomycin (P/S) (Thermo Fisher Scientific, cat. no. 15140122), 2 mM GlutaMAX (Thermo Fisher Scientific, cat. no. 35050061), 1% NEAA (Thermo Fisher Scientific, cat. no. 11140050), 0.1% β-mercaptoethanol (Thermo Fisher Scientific, cat. no. 21985023). The Marc145 were passaged every 3 days and cultured at 37 °C/5% CO_2_, under normoxia conditions, in STO medium. All cell lines were free from *mycoplasma* and were detected by PCR.

### Differentiation of macrophage from pEPSCs

The differentiation medium contains StemSpan SFEM II (StemCell Technologies), 1% penicillin/streptomycin (P/S) (Thermo Fisher Scientific, cat. no. 15140122), 2 mM GlutaMAX (Thermo Fisher Scientific, cat. no. 35050061), 1% NEAA (Thermo Fisher Scientific, cat. no. 11140050), 0.5% N2 supplement (Thermo Fisher Scientific, cat. no. 17502048), 1% B27 supplement (Thermo Fisher Scientific, cat. no. 17502048). pEPSCs were passaged as normal then preplated on a 0.1% gelatinized 35 mm for 10 min at 37  °C/5% CO_2_ to remove feeder cells. Floating pEPSCs were collected and resuspended in the differentiation medium supplemented with 25 ng/ml BMP4 (PeproTech, cat. no.120-05ET), 25 ng/ml Activin A (Sino Biological, cat. no. 10429-HNAH), and 6 μM CHIR99021 (MedChemExpress, cat. no. HY-10182) for the first 2 days (day 0 to day 2). On day 2, cells were refreshed with the differentiation medium supplemented with 50 ng/ml VEGFA (Sino Biological, cat. no.11066-HNAH), 50 ng/ml bFGF (Sino Biological, cat. no. 10014-HNAE), 50 ng/ml SCF (Novoprotein, cat. no. C034), and 10 μM SB431542 (MedChemExpress, cat. no. HY-10431). On day 5 and day 7, cells were refreshed with the differentiation medium supplemented with 50 ng/ml VEGFA, 50 ng/ml bFGF, 50 ng/ml SCF, 50 ng/ml IL-6 (Novoprotein, cat. no. C009), 50 ng/ml feline McDonough sarcoma-related tyrosine kinase 3 ligand (Novoprotein, cat. no. CA82), 50 ng/ml thrombopoietin (Novoprotein, cat. no. CJ95), and 10 ng/ml IL-3 (Novoprotein, cat. no. CX90). In this process, a large number of suspended EMPs were released into the supernatant. On day 9, floating cells were collected and resuspended in differentiation medium supplemented with 80 ng/ml macrophage colony-stimulating factor 1 (Novoprotein, cat. no. C417). After 2 days, all EMPs differentiated into macrophages. Cells were cultured on 24-well culture plate, at 37 °C/5% CO_2_, under normoxia conditions throughout the differentiation.

### Giemsa staining

The pPSCdMs and PAMs were cultured as normal and then fixed with 70% ethanol for 10 min. Adding 1× modified Giemsa staining solution (Beyotime, cat. no. C0131) to the 24-well tissue culture plate to stain for 15 min then washing twice with ddH_2_O according to the manufacturer's instructions.

### RT-qPCR

Total RNA was extracted using RNAiso Plus reagent (Takara, cat. no. 9108) by the method of guanidine isothiocyanate phenol–chloroform and the quality of the extracted total RNA was detected by Nanodrop spectrophotometer (Thermo Fisher Scientific Inc). Complementary DNA (cDNA) was synthesized from 2 μg RNA using HiScript III RT SuperMix for qPCR (+gDNA wiper) (Vazyme, cat. no. R323) according to the manufacturer's instructions. RT-qPCR was performed by using ChamQ SYBR qPCR Master Mix (Vazyme, cat. no. Q311) in 3-step process. The primers used for RT-qPCR were designed by Primer Premier 5 and shown in Table S1.

### Immunofluorescent staining

The pPSCdMs and PAMs were cultured as normal, and then fixed with 4% paraformaldehyde (pH 7.4) at room temperature for 15 min after cells were washed three times with PBS. The pPSCdMs and PAMs were blocked in 10% FBS at room temperature for 1 h and incubated with primary antibodies at 4 °C for 12 h, then washed three times with PBS. The primary antibodies including CD14 (Biorad, cat. no. MCA1218F, 1:200), CD163 (Biorad, cat. no. MCA2311F, 1:200), CD45 (Biorad, cat. no. MCA1746GA, 1:200). Finally, cells were incubated with goat anti-mouse IgG (H + L) secondary antibody alexa fluor 488 conjugate (1:500; cat. no. ZSGB-BIO) at room temperature for 1 h and cell nuclei were stained with Hoechst 33342 (Sigma-Aldrich, cat. no. B2261, 1:1000) at room temperature for 5 min. The fluorescent images were taken with EVOS M5000 microscope (Thermo Fisher Scientific, cat. no. AMF5000).

### PRRSV infection

The pPSCdMs, PAMs, and Marc145 were cultured as normal and incubated with GFP-labeled PRRSV (GFP-PRRSV) for 12 h (66). The supernatant cultures of pPSCdMs and PAMs at 72 h after PRRSV infection were collected for secondary infection. The fluorescent images were taken with EVOS M5000 microscope (Thermo Fisher Scientific, cat. no. AMF5000).

### PI staining

The PRRSV infected pPSCdMs and PAMs were incubated with 6.7 μg/ml PI (Solarbio, cat. no. C0080) for 5 min. The pPSCdMs and PAMs were washed three times with PBS. The dead cell nuclei were stained with PI and presented red fluorescence. The fluorescent images were taken with EVOS M5000 microscope (Thermo Fisher Scientific, cat. no. AMF5000).

### LPS induction

The pPSCdMs and PAMs were cultured as normal and incubated with LPS (200 ng/ml, from *E. coli* O111:B4, Sigma-Aldrich, cat. no. L2630) for 24 h. The expression of related-genes was detected by RT-qPCR.

### Flow cytometry analysis

Cells were dissociated to form single cell-suspensions with 0.05% trypsin/EDTA (Thermo Fisher Scientific, cat. no. 25300062). Then, cells were washed once with fluorescence-activated cell sorting buffer and stained with antibodies for 30 min at 4 °C. Samples were analyzed on CytoFLEX (Beckman Coulter). The results were analyzed using CytExpert. Antibodies used were KDR (Proteintech, cat. no. 26415-1-AP, 1:100), CD31 (Biorad, cat. no. MCA1222GA, 1:100), CD45 (Biorad, cat. no. MCA1746GA, 1:100), and CD163 (Biorad, cat. no. MCA2311F, 1:100).

### Endocytosis assay

The pPSCdMs and PAMs were cultured as normal in 0.1% gelatinized 24-well tissue culture plate. Then, the pPSCdMs and PAMs were incubated with 1 mg/ml tetramethylrhodamine-dextran (Thermo Fisher Scientific, cat. no. D1818) cell culture incubation medium at 37 °C/5% CO_2_ for 30 min (52). The pPSCdMs and PAMs were washed five times with ice-cold PBS then fixed with 4% paraformaldehyde (pH 7.4) at room temperature for 15 min. Cell nuclei were stained with Hoechst 33342 (Sigma-Aldrich, cat. no. B2261, 1:1000) at room temperature for 5 min. The fluorescent images were taken with Laser scanning confocal microscope (Laser, cat. no. TCS SP8 SR) and positive dextran uptake was shown in red. The macropinocytic index is the percentage of dextran-positive cells area accounting for the total cells area during the same dextran uptake time.

### Phagocytosis assay

The pPSCdMs and PAMs were cultured as normal in 0.1% gelatinized 24-well tissue culture plate. Then, the pPSCdMs and PAMs were incubated with 1 mg/ml pHrodo Green *E. coli* BioParticles Conjugate (Thermo Fisher Scientific, cat. no. P35366) cell culture incubation medium at 37 °C/5% CO_2_ for 3 h following the manufacturer's instructions. The pPSCdMs and PAMs were washed three times with PBS then fixed with 4% paraformaldehyde (pH 7.4) at room temperature for 15 min. Cell nuclei were stained with Hoechst 33342 (Sigma-Aldrich, cat. no. B2261, 1:1000) at room temperature for 5 min. The fluorescent images were taken with EVOS M5000 microscope (Thermo Fisher Scientific, cat. no. AMF5000) and positive green *E. coli* BioParticles uptake were shown in green. Phagocytic index is the percentage of positive macrophages multiplied by their mean of fluorescence and is normalized on 20 μM cytochalasin (MedChemExpress, cat. no. HY-N6774) treated samples (67).

### RNA-seq

Total RNA was extracted using RNAiso Plus reagent (Takara, cat. no. 9108) according to the manufacturer's instructions. The quality of the extracted total RNA was detected by NanodropTM spectrophotometer (Thermo Fisher Scientific Inc) and agarose gel electrophoresis. Then, mRNA was purified from 2 μg total RNA by using Dynabeads Oligo (dT) (Thermo Fisher Scientific) with two rounds of purification and fragmented with divalent cations under elevated temperature. Then the cleaved RNA fragments were reverse-transcribed to create the cDNA by SuperScript II Reverse Transcriptase (Invitrogen, cat. no. 1896649), which were next used to synthesize U-labeled second-stranded DNAs with *E. coli* DNA polymerase I (NEB, cat. no. m0209), RNase H (NEB, cat. no. m0297) and dUTP Solution (Thermo Fisher Scientific, cat. no. R0133). After the heat-labile UDG enzyme (NEB, cat. no. m0280) treatment of the U-labeled second-stranded DNAs, the ligated products were amplified with PCR. The average insert size for the final cDNA librarys were 300 ± 50 bp. At last, we performed the 2 × 150 bp paired-end sequencing (PE150) on an Illumina NovaSeq 6000 (LC-Bio Technology CO, Ltd) following the vendor's recommended protocol.

### Bioinformatics

We aligned reads of all samples to the pig reference genome (Sscrofa11.1) (68) with HISAT2 (69) (https://daehwankimlab.github.io/hisat2/, version:hisat2-2.2.1) package, which allows multiple alignments per read up to 20 (by default) and a maximum of two mismatch when mapping the reads to the reference. Mapping rates were consistently above 90%. The mapped reads from each sample were assembled using StringTie (70, 71) (http://ccb.jhu.edu/software/stringtie/, version: stringtie-2.1.6) with default parameters. Once the final transcriptome was generated, StringTie was used to estimate the expression levels of all transcripts. R (http://www.r-project.org/) was used to perform correlation analysis and PCA. The Pearson correlation coefficient between two replicas was calculated to evaluate repeatability between samples. PCA was performed with the prcomp function of R. Differential gene expression analysis was performed by DESeq2 (72) (https://www.bioconductor.org/packages/release/bioc/html/DESeq2.html) software between pEPSCs and other groups. The genes with the parameter of false discovery rate below 0.05 and an absolute fold change ≥ 1 were considered DEGs (73). DEGs were then subjected to GO (74) enrichment analysis and KEGG (75) enrichment analysis using the website DAVID (76, 77) (https://david.ncifcrf.gov/).

### Statistical analysis

Two-tailed *t*-tests were used to determine significant differences between the two groups. All data are shown as mean ± sd. Differences were considered significant when *p* < 0.05.

## Data availability

All relevant data can be found within this article and its supporting information (SRA Accession: PRJNA1037615). Porcine alveolar macrophages RNA-seq data (58, 59) is obtained from the NCBI GEO database (GSE172284) and the NCBI GEO database (GSE145954). Microglia RNA seq data (58) is obtained from the NCBI GEO database (GSE172284). Peripheral blood mononuclear cells RNA seq data is obtained from the NCBI GEO database (GSE116701). Pig RNA-seq datasets of PSCdM cell lines from Meek Lab (42) is obtained from the NCBI (BioProjcet accession: PRJNA787759). Parental porcine PSC lines (44) are available from Yiliang Miao (miaoyl@mail.hzau.edu.cn) and Pentao Liu (pliu88@hku.hk).

## Supporting information

This article contains supporting information (10, 28, 45, 46, 54, 67).

## Conflict of interest

The authors declare that they have no conflicts of interest with the contents of this article.
