# Supplementary material for: Rapid conversion of porcine pluripotent stem cells into macrophages with chemically defined conditions
Source: J Biol Chem. 2023 Dec 12;300(1):105556. doi: 10.1016/j.jbc.2023.105556 (PMC10825052; doi:10.1016/j.jbc.2023.105556)
Supplement: Supporting Table S1 [file mmc1.docx]

Supporting information of table.

Table 1 The primers used for qRT-PCR

| Gene | Forward Primer | Reverse Primer |
| --- | --- | --- |
| β-ACTIN | GCAAGGACCTCTACGCCAACA | TGGAGGCGCGATGATCTTG |
| OCT4 | CTTCACCACCCTGTACTCCTCG | CAGGCTTCTCTCCCTAGCTCAC |
| SOX2 | ATGTCCCAGCACTACCAGAGCG | CTTACTCTCCTCCCATTTCCCTCT |
| NANOG | CTTCACCAATGCCTGAGGTTTATG | AGGGCTGTCCTGAATAAGCAGATC |
| EOMES | ACATGCAGGGCAACAAAATGTATG | TGAAGGTCTGAGTCTTCGAGGG |
| TBXT | GCCAGATCATGCTGAACTCCTTA | ATAAGCCGTCACCGCTATGAAC |
| MIXL1 | CTTCCGCAGGACCATGTACC | GCTTCAAATATCTCCCTTCCGTTTC |
| KDR | CATTGAGTCCAATCACACAATCAAAG | CATTCACAACCAGAGATACCACG |
| HNF4A | TGGTGGACAAAGACAAGAGGAAC | GCTATCCTCGTAGCTTGACCTG |
| CD31 | CAGAAGGGAGGCATGATGATG | AAGTTGGTAGGAAATGGGCGA |
| CDH5 | AGAAGAACCTGGAGTCTCCTTCC | TCATCTGCGTCTACTGCTGTCA |
| CD34 | CTCTGCTTGCTGAGTTTGCTG | ATCTGGTGTGGTTTCCTCGTTG |
| RUNX1 | GCAACTGCAGCCATGAAAAACC | AGCCGCTCGGAAAAGGACAA |
| CD41a | AAGAACGAGGAGGCTGAGAAGA | CCGTGCCATGAGACCTAAGAAAT |
| CD45 | GACATCAATAGCCCTGCTTGTAGT | GCGATCTTTCTCTTGTAAGTTTCCAAC |
| CD43 | CACCATTGAAACTGGCTTGCT | TCTGACCTGAATTTTGGGGGG |
| CD14 | CCCTGAGCTCTCTCAATTTGTCAT | CAGGATCCATATAGGGATTCCCTTC |
| CD163 | GGAGTTGCCCTTTCTATCCCG | AGCAGATTACAGAGGCCACTTG |
| SPI1 | CGTGCAAAATGGAAGGGTTTCC | GTGAAGTGGTTCTCGGCGAA |
| CSF1R | CAACAATCCGACTTCCACGAC | TTCTCTCCCACTGCTACCTCC |
| CSF2RB | TATCCGCACATAGGTTACATCTTCC | GACTGCTCCTTACTCCACTTGC |
| CSF2RA | AGATACAACCCTCCCAACAACAT | CCAAATCACCAGACACCACAAT |
| DDX58 | ATCCAAACCAGAGGCAGAGG | TCTTTGTCGATCAGATCAGCG |
| TNF | ACGGCGTGAAGCTGAAAGAC | GAGTGAGGAAAACGTTGGTGGAA |
| IL6 | TGTCGAGGCTGTGCAGATTAGTA | CATTTGTGGTGGGGTTAGGGGT |
| IL10 | CTTGTTGCTGACCGGGTCTCT | CTCCCCATCACTCTCTGCCTT |
| IL1A | AACGAAGACGAACCCGTGTTG | GATGGGCGGCTGATTTGAAGT |
| IL1B | GTGACCTTAGGGATCAAGGGAAAG | GTTGGGGTACAGGGCAGAC |
| CCL4 | CACCTCCTGCTGCTTCACATACA | CTCCTGGACCCAGTCATCACTG |
| GBP1 | GCGACCAAACTAGACAAAAAGCG | CGACGTAGAGACGATACCCTCC |
| MRC1 | AAGGTGGATTATGTGGCTTGGG | GTATGGTGGGGGTAACTGTGG |
| CD68 | TTGGCTGTGCTCTTCTTAGGG | AGGATTGTGAGTGACTGTTGTGG |
| ITGAM | TGATGGACTTGGCTGTAGGGG | CGAACTTGGAGGCAGACTTGG |
| CD86 | GGATGAGCTGGTCATATTTTGGC | CCTTGATTTGAACGTTGTGGAGTC |
| NUP62 | AGCTCGACTTCATCCTGTCTCAG | TAGGTCTTCTCTCGCTCCTCATC |
